# Micellization of Zwitterionic Surfactant with Opposite Dipoles is Differently Affected by Anions

**DOI:** 10.1021/acs.langmuir.5c00360

**Published:** 2025-03-31

**Authors:** Laura Mortara, Matheus P. Cortez, Caroline D. Lacerda, Gustavo P. B. Carretero, Shirley Schreier, Hernan Chaimovich, Filipe S. Lima, Iolanda M. Cuccovia

**Affiliations:** † Instituto de Química, Universidade de São Paulo, São Paulo 05513-970, Brazil; ‡ Departamento de Química Fundamental, Centro de Ciências Exatas e da Natureza, Universidade Federal de Pernambuco, Recife 50670-901, Brazil

## Abstract

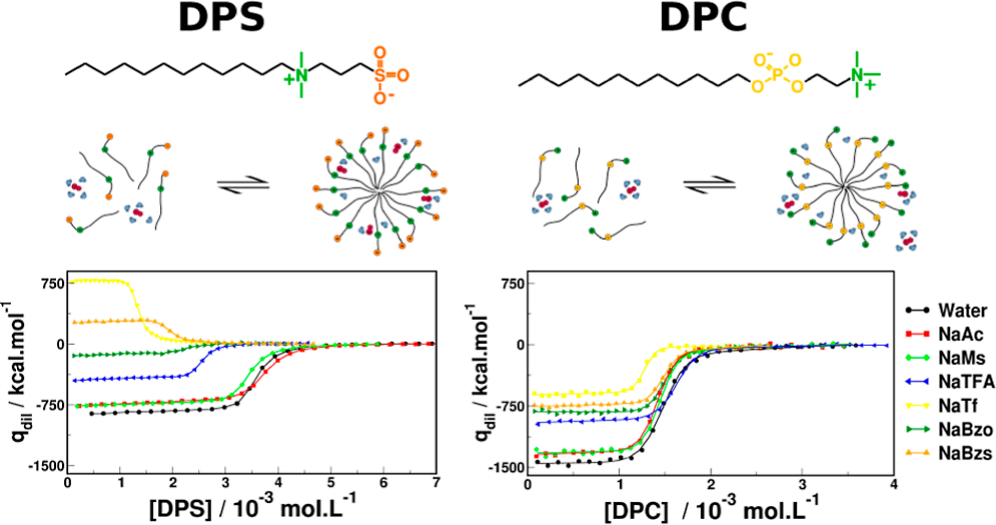

We used isothermal titration calorimetry to investigate the influence of hydrotropic anions on the micellization of the zwitterionic surfactants *N*-dodecylphosphocholine (DPC) and *N*-dodecyl-*N*,*N*-dimethyl-3-ammonio-1-propanesulfonate (DPS), as well as the effect of these anions on the micellar interface. Our results showed an increased enthalpic contribution to the Gibbs energy for micellization when the hydrotropic anions contained CF_3_ or C_6_H_5_ hydrophobic groups. We found that the critical micelle concentration of DPS was more sensitive to the effects of the anions than that of DPC, likely because the positive charge of DPS is located closer to the micellar core. Overall, the magnitudes of the anion effects on the micellar properties of DPC and DPS were relatively minor compared with those observed in cationic micelles. These findings offer valuable insights into the role of dipole orientation and charge distribution in the interactions between zwitterionic surfactants and anions as well as the effects of hydrotropic anions on these systems.

## Introduction

Above a particular concentration called the critical micelle concentration (CMC), surfactants form micellar aggregates.^1^ The transfer of the hydrophobic moiety of the surfactants to the micellar apolar core contributes favorably to micelle formation. In contrast, the reduction in available configurations for these aggregated amphiphilic molecules, caused by the spatial constraint of hydrophobic chains within the micelle core and the confinement of head groups to the micelle surface, imposes an entropic cost that limits micelle growth,^2^ together with repulsion forces between the charged surfactant head groups.^3^ Like oil–water separation, micelle aggregation depends on the length scale, i.e., aggregation occurs when small molecules entropy-dominated solvation free energy surpasses the enthalpy-dominated solvation free energy of larger surfaces.^4^ It is also known that a significant heat capacity change is observed for processes involving transferring nonpolar molecules from water to aggregates, such as oil–water phase separation and protein and surfactant aggregation. A two-state model, based on the enthalpy difference arising from the number of hydrogen bonding reduction due to the presence of interface, explains the heat capacity changes in the solvation shell.^5^

CMC and, consequently, Gibbs free energy of micellization show a nonmonotonic behavior with temperature for several ionic,^6^ nonionic,^7^ and zwitterionic^8^ surfactants, like the free energy of transfer for hydrophobic molecules.^4,9^ In contrast, the enthalpy of micellization is endothermic at lower temperatures and becomes exothermic as temperature rises.^10^

Salt effects on the CMC depend on the concentration and the nature of added ions,^10,11^ and the explanation for specific ion effects relies on salt-induced alterations in water properties or direct chemical binding.^12^ In ionic micelles, counterion interactions with the charged headgroup lead to lower interfacial electrostatic repulsion at the interface and, consequently, a lower CMC.^13,14^ Larger and less hydrated ions like Cs^+^ or Br^–^ diminish the CMC to a higher degree than small hydrated ions like Li^+^ and F^–^ in negatively alkyl sulfates^10^ or positively charged akyltrimethylammonium^11^ surfactants, for example. For nonionic surfactants, the salt effect on micellization is usually interpreted as a salting-in and salting-out phenomenon, explained by the lower solubility of hydrophobic molecules induced by the presence of salts in the aqueous solution, with the role of salt-headgroup interaction considered minor in this case.^15−17^

Zwitterionic surfactants (ZwS) are electrically neutral, containing positive and negative charges in their headgroup. ZwS are usually highly water-soluble, exhibit low toxicity,^18^ and are less irritating to the skin and eyes than ionic ones.^1^ Due to these properties, ZwS are constituents of cosmetics and are suitable for medical applications, such as nanoemulsion-based vaccines^19,20^ and used for protein solubilization.^21^ Although ZwS aggregates have zero net charges, ion adsorption into the dipolar region can screen the charges and decrease the CMC,^22^ as observed for ionic surfactants. Still, the CMC decrease can also be attributed to salt-induced modifications in water properties,^23^ as observed for nonionic surfactants. Also, although ion effects are observed in ZwS micelles, this effect is milder when compared with ionic surfactants.^24^

As pointed out above, ion hydration, size, and charge are some of the factors responsible for the ion-specific effects. Still, the nature of the interface also needs to be considered. For instance, other properties such as ion-paring, complexation capacity, and ion hydrophobicity are also important to assess the specific interaction of ions with surfactants.^25^ Collin’s law of matching water affinities has been used to explain surfactant-ion pairing,^26^ focusing solely on the formation of contact ion pairs due to similarity in the sizes of interacting groups. Hydrotropes are relatively small anions and, as surfactants, have a hydrophilic and a hydrophobic portion in their structure but, due to their size, do not form aggregates in solution.^27^ Due to this characteristic, these anions have an affinity for the aggregates’ interface and hydrophobic interior, strongly partitioning in interfaces of cationic micelles.^28,29^ We have shown the effect of hydrotropes in cationic surfactants, where anions like trifluoromethanesulfonate (triflate, Tf) have high adsorption at the interface and induce micellar growth and even phase separation.^30^ Addition of cationic drugs containing hydrophobic portions significantly lowered CMC for anionic surfactants.^31^ Thus, the thermodynamics of micellization of aggregates containing hydrotropic ions may differ from other ions due to more terms that contribute to the energy balance of micelle formation, such as hydrophobic interactions and dehydration.^29,32,33^

The CMC of dodecyl dimethylammonium propanesulfonate (DPS) surfactant decreases significantly after adding salts containing a hydrophobic anion.^34^ Further studies on the effect of hydrotropic anions on ZwS micellization, despite the importance of these additives, are scarce. The adsorption of anions in ZwS with opposite dipoles has also been studied, but the number and the structure of the different anions are limited.^35,36^

Isothermal titration calorimetry (ITC) is an adequate tool for studying ion-specific effects on the thermodynamics of micellization because the CMC and enthalpy of the process can be measured precisely. Here, we evaluated how the thermodynamics of micellization of two ZwS, *N*-dodecylphosphocholine (DPC) and *N*-dodecyl-*N*,*N*-dimethyl-3-ammonium-1-propanesulfonate (DPS), are affected by some sodium salts of perchlorate (NaClO_4_), trifluoroacetate (NaTFA), trifluoromethanesulfonate (NaTf), benzenesulfonate (NaBzs), benzoate (NaBzo), chloride (NaCl), bromide (NaBr), methanesulfonate (NaMs), and acetate (NaAc). These anions were chosen to evaluate the effect of dipole reversal upon ion binding and the influence of the “hydrophobic” group of the anions on micellar properties.

## Materials and Methods

### Materials

DPC (Avanti >99%) was used as received. DPS (Sigma ≥ 97%) was recrystallized 2× from acetone/methanol. Sodium bromide (Synth ≥99%), chloride (Synth ≥99%), perchlorate (Merck), benzoate (NaBzo) (Sigma-Aldrich >99%), and acetate (NaAc) (Baker ≥99.5%). Sodium methanesulfonate (NaMs) (Sigma ≥98%) was washed 2× with acetone. Sodium trifluoroacetate (NaTFA), trifluoromethanesulfonate (NaTf), and benzenesulfonate (NaBzs) were synthesized by the neutralization (NaOH) of respective acids (trifluoroacetic acid—Sigma-Aldrich ≥98%; trifluoromethanesulfonic acid—Sigma-Aldrich ≥98%; benzenesulfonic acid—Sigma-Aldrich ≥98%). Methyl 5-doxyl-stearate (5-MeSL) (Sigma-Aldrich 100%) was used as received.

### Methods

#### Isothermal Titration Calorimetry

ITC was performed with VP-ITC (Microcal Inc.). Solid DPS and DPC were dissolved in aqueous solutions containing no salt or 0.1 M of either NaAc, NaBr, NaCl, NaMs, NaTFA, NaBzs, NaBzo, NaTf, or NaClO_4_. The surfactant concentration in the addition syringe was 12 times the CMC (∼0.04 M for DPS and ∼0.02 M for DPC). Different saline solutions without surfactant were placed in the calorimeter cell, and the surfactant, dissolved in the same saline solutions, was injected stepwise into this solution with the ITC syringe (32 injections of 8 μL each). The time between injections (300 s) was enough for the signal to return to baseline values. Upon each injection, a peak was produced, and a plot containing the integration of each heat peak as a function of surfactant concentration was constructed. A modified sigmoidal expression (eq 1)^37^ was used to fit the *q*_dil_/surfactant dependence.
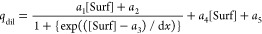
1where *a*_i_’s are fitting parameters and [Surf] is the concentration of the surfactant in the cell after each addition. The CMC was taken as the maximum (or minimum) of the first derivative of the adjusted equation, and the standard demicellization enthalpy (Δ*H*_demicellization_^°^) was taken as the difference between the two baselines at the CMC, one at the beginning and the other one at the end of the titration. In the Results section, we report standard enthalpy of micellization, i.e., (−1) × Δ*H*_demicellization_^°^.

The standard Gibbs energy, Δ*G*^°^, was determined from the CMC.^38^ For nonionic or ZwS, CMC can be approximated as (eq 2)^39^
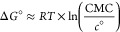
2where *R* is the gas constant, *T* is the temperature in kelvin, CMC is the critical micelle concentration, in mol L^–1^, and *c*° is the standard concentration. For ionic micelles, counterion association is included,^40^ but due to the low counterion association in zwitterionic micelles,^41^ we used the simplified version in eq 2 to extract Δ*G*° of micellization, and with Δ*H*° determined from the fit in eq 1, Δ*S*°, the entropy change, can be determined using eq 3.
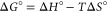
3

## Results and Discussion

**Figure 1:**
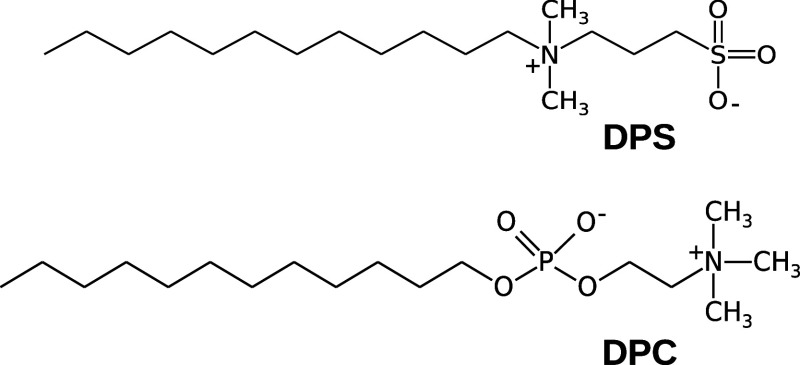
Chemical structures of DPS (up) and DPC (below).

DPC and DPS are ZwS with a hydrocarbon chain length of 12 carbons. The positive charge on the quaternary nitrogen in the DPS monomer headgroup is directly bound to the hydrocarbon chain (Figure 1). In contrast, in DPC, the negatively charged phosphate group is directly bound to the hydrocarbon chain, resulting in the two surfactants having opposite dipoles with respect to the hydrophobic chain. The negatively charged groups are different, with DPS bearing a sulfate anionic group, which is more chaotropic than the monovalent phosphate group^26^ in DPC. Nevertheless, it is well-known that anions adsorb more readily than cations in zwitterionic micelles.^42^ Because both surfactants in this study possess a similar positively charged group, the anionic region of the surfactant is expected to play a minor role in ion adsorption at the micellar interfaces.

**Figure 2:**
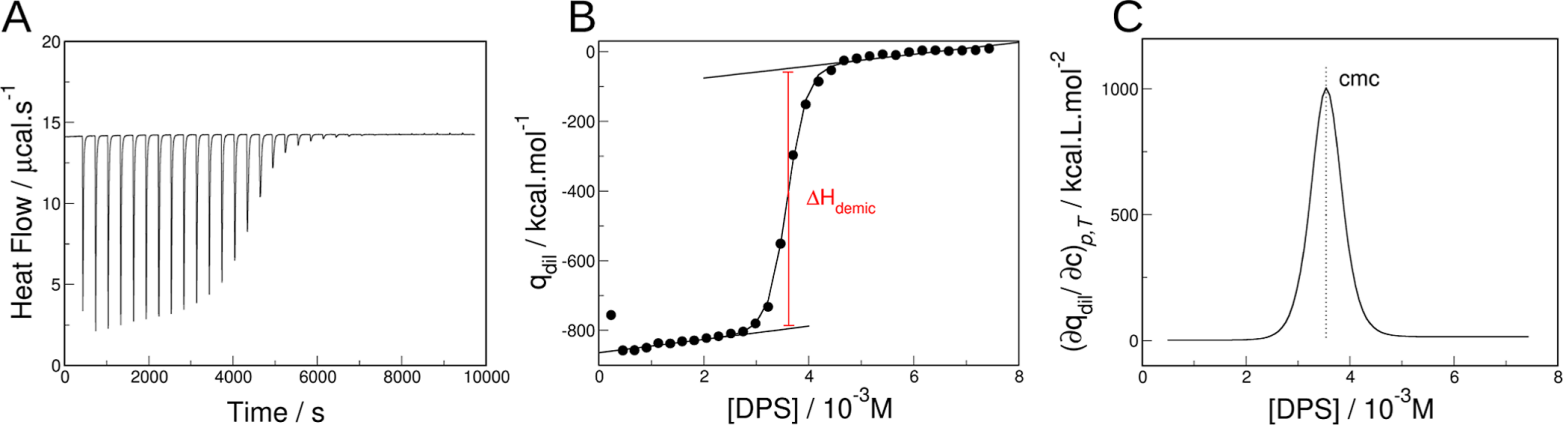
Titration calorimetry experiment for DPS in water at 25 °C. (A) Heat flow versus time upon 32 sequential injections of 8 μL of 0.04 M DPS into 1.4 mL of water at 25 °C. (B) Integration of peak area (points) as a function of the DPS concentration ([DPS]), fitting with eq 1 and determination of Δ*H*_demicellization_. (C) The first partial derivative of the adjusted equation presented in (B), CMC is determined at the peak maximum.

In Figure 2, we show a typical ITC experiment showing the effect of the sequential addition of a stock solution of 0.04 M DPS to water and the corresponding data fitting.

**Figure 3:**
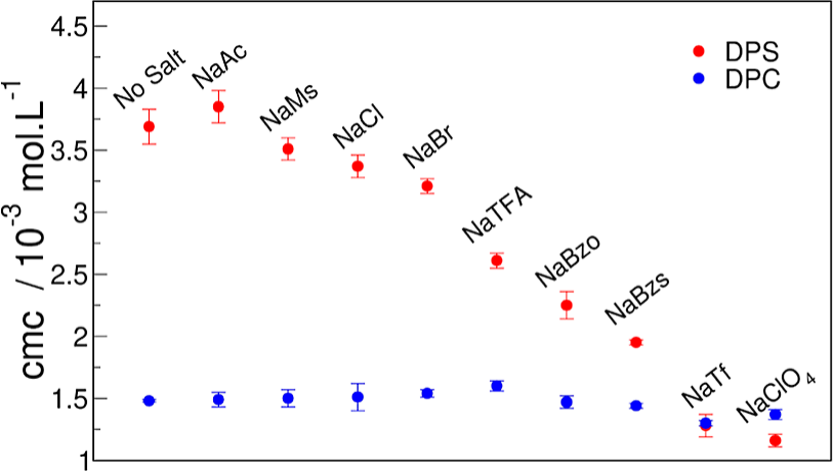
CMC for DPS (●, red) and DPC (●, blue) at 25 °C in 0.1 M of salts.

The effects of anions on the micellization thermodynamics of DPS and DPC were determined by ITC using 0.1 M salt solutions. Since the trends observed for both CMC and enthalpy of micellization persist at other salt concentrations for DPS (not shown), a single concentration was sufficient to compare the effect between both surfactants. Titration calorimetry in the presence of salts is similar to the data in Figure 2 (not shown). CMCs and enthalpy of micellization were measured at 10 °C, 25 °C, and 35 °C for DPS and 15 °C, 25 °C, and 35 °C for DPC. Figure 3 shows the calculated CMCs for DPS and DPC at 25 °C in 0.1 M salt solution of NaAc, NaMs, NaCl, NaBr, NaTFA, NaBzo, NaBzs, NaTf, and NaClO_4_. The CMCs at the other measured temperatures are in the Supporting Information (Tables S1 and S2).

The CMC for DPS in water was 3.6 mM at 25 °C, consistent with previous data.^8,34^ Salt addition, except for NaAc, decreased the CMC of DPS. DPS micellization was salt-dependent and critically dependent on the nature of the anion, as the CMC decreased in the presence of some salts by a factor of almost four (Figure 3). The value of CMC followed the order NaAc ∼ NaMs ∼ NaCl > NaBr > NaTFA > NaBzo > NaBzs > NaTf ∼ NaClO_4_, very similar to the counterion effect on the micellization of 1-dodecyl-3-methylimidazolium ([C_12_mim]^+^)^43^ and dodecyltrimethylammonium (DTA^+^),^29^ both cationic surfactants. The CMC of CHAPS, 3-[(3-cholamidopropyl) dimethylammonio]-1-propanesulfonate, a ZwS containing a similar headgroup as DPS, is also reduced upon NaCl addition but only at considerably higher salt concentrations (0.5 M).^24^

For DPC, CMC in water was 1.5 mM, higher than previously determined by different methods^44,45^ but similar to that determined by ITC.^46^ Within experimental error, the effect of salts on the CMC of DPC within experimental error was negligible regardless of the nature of the salt. Previous studies on salt effects of DPC using a higher salt concentration (∼0.45 M) show that the addition of hydrophobic anions like ClO_4_^–^ lowered the CMC,^44^ while anions like Cl^–^ only affect CMC at even higher concentrations (1.45 M), but cation effect is observed in smaller concentrations (0.1 M).^44^

Although DPS and DPC are both ZwS, DPS’s positive charge is closer to the molecule’s apolar moiety (Figure 1). Kinetic decarboxylation results show that the interface of DPS is more dehydrated than DPC,^47^ and chemical trapping also shows that anion adsorption at the micellar interface is smaller for phosphocholine surfactant.^35^ Notably, the headgroup orientation toward the micelle surface interferes with the anion effect. A tilt in the headgroup toward charge neutralization was observed in both surfactants in water due to the presence of both charges in the headgroup of the surfactant, using molecular dynamics simulation for DPS^48,49^ or DPC,^50^ and also experimentally inferred by NMR for phosphocholine lipids.^51,52^ Headgroup extension in the presence of anions like ClO_4_^–^ is observed for DPS using MD^48,49^ and for phosphocholine lipids experimentally.^53^

Because ZwS are formally neutral, ion effects could be similar to nonionic surfactants.^54^ The decrease in the CMC with added salt is attributed to indirect effects, such as the salting-out of the hydrocarbon moiety of the monomeric surfactant molecule. Strongly hydrated anions like Cl^–^ diminish the solubility of monomers, lowering the CMC more effectively than the weekly hydrated anions like ClO_4_^–^.^17,55,56^ This is the opposite of what we observed here for both ZwS, where a more significant CMC decrease was produced by NaClO_4_. Anion adsorption in zwitterionic micelles was observed in molecular dynamics simulations^48,49^ and experimentally, using ion-specific electrodes^41^ and zeta potentials measurements, that showed negative values upon salt addition in otherwise neutral surfactants.^57^ These results indicate that the effect of salts on ZwS aggregation is a direct effect related to ion binding at the micellar interface and must be compared with ionic rather than nonionic surfactants, where the headgroup repulsive interactions are shielded by electrolytes, leading to a decrease in the CMC.^42^

**Figure 4:**
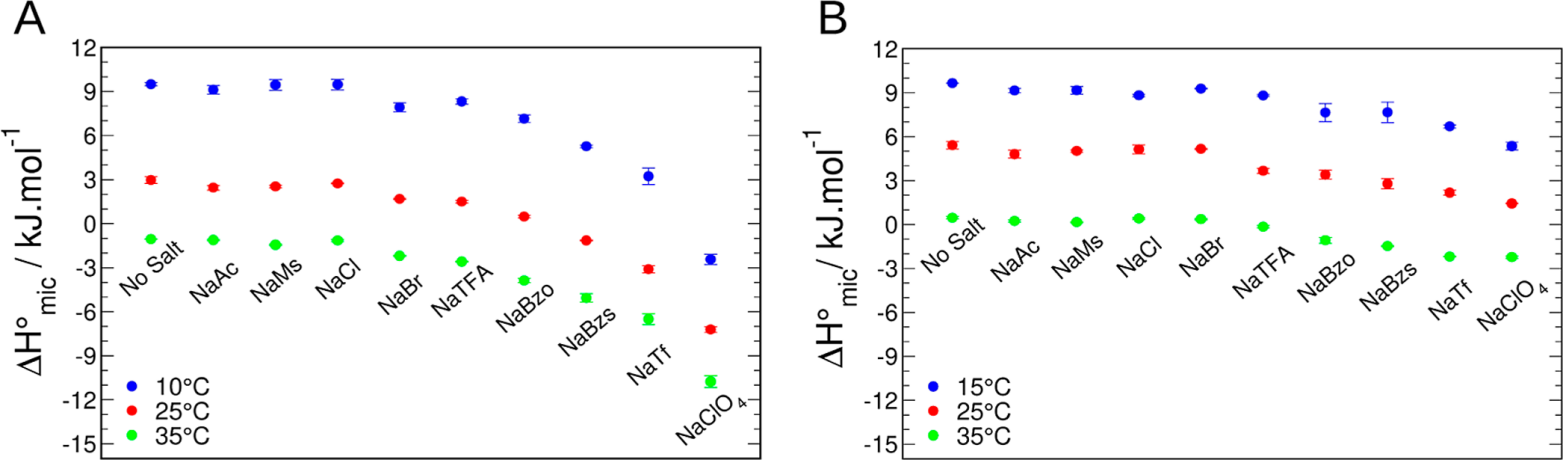
Enthalpy of micellization for DPS and DPC in solutions containing 0.1 M of the salts indicated in the figures: (A) DPS at 10 °C (●, blue), 25 °C (●, red), and 35 °C (●, green) and (B) DPC at 15 °C (●, blue), 25 °C (●, red), and 35 °C (●, green).

With ITC, we determined the micellization enthalpies for both surfactants at three different temperatures using ITC (Figure 4).

For both surfactants, the decrease in the enthalpy of micellization with added salt follows the same order as for the CMC. Salt effects were more pronounced in DPS than in DPC. The main energetic contributions of the transfer of surfactant monomers from the water to the micelle are the release of hydration water from the surfactant and the repulsion of surfactant headgroups—which can be screened by ions.^10^ Enthalpy of transferring nonpolar molecules from water to the apolar phase is correlated with creating a water/oil interface, which involves breaking hydrogen bonds between water molecules at this interface.^4,5^ For amphiphilic molecules like surfactants, there are also contributions from the headgroup. Since micelles studied here have similar sizes due to similar aggregation numbers,^34^ significant enthalpy differences must arise from contributions within the polar headgroup region. At a fixed salt concentration, the decrease of Δ*H*° follows qualitatively the degree of counterion condensation for cationic imidazolium-based^43^ surfactant, suggesting that the screening of repulsive charges at the headgroup region is responsible for the selective ion effect reported here. It should be noted that the binding of perchlorate in sulfobetaine micelles is exothermic.^58^

The micellization process was endothermic at low temperatures and became exothermic at higher temperatures, reflecting this process’s negative heat capacity change, Δ*C*_p_,_mic_, characteristic of the hydrophobic effect.^59^ For most surfactants micellization and nonpolar molecules solubilization,^60,61^ the temperature at which the enthalpy change is zero (*T*_H_) is typically near room temperature (298 K).^24^ When NaClO_4_ was added to DPS, *T*_H_ = 276 K, significantly lower compared to the other analyzed salts, which are around 300 K (Table S3). Reduction of *T*_H_ was also observed for NaTf (*T*_H_ = 291 K). A similar reduction has been reported for a cationic surfactant with iodide or salicylate as counterions, both of which exhibit a high degree of counterion adsorption and for anionic alkyl sulfate surfactant with cesium as a counterion.^10,43^ Thus, this effect on enthalpy is probably associated with a higher degree of ion adsorption at the zwitterionic interface for both perchlorate and triflate.

As stated above, heat capacity change, Δ*C*_p,mic_ reflects the enthalpy variation with temperature. For solubilization of nonpolar molecules in water, the heat capacity change (Δ*C*_p_) between gaseous and dissolved states is correlated with the molecular surface area and the reorganization of water hydrogen bonds in the first hydration shell can explain the magnitude of Δ*C*_p_.^5^ Thus, Δ*C*_p_, is often calculated for micelle formation.^10,24,62−64^ For nonionic surfactant aggregation, Δ*C*_p,mic_ is directly proportional to the removal of the water-accessible nonpolar surface area.^63,64^ Linear increases of Δ*C*_p,mic_ magnitude with hydrocarbon tail size is also often observed for ionic surfactants with the same headgroup and counterion.^43,62^ In the temperature range considered here, we assume that the enthalpy of micellization scales linearly with temperature, allowing us to extract the heat capacity changes (as shown in eq 4).
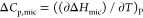
4

**Figure 5:**
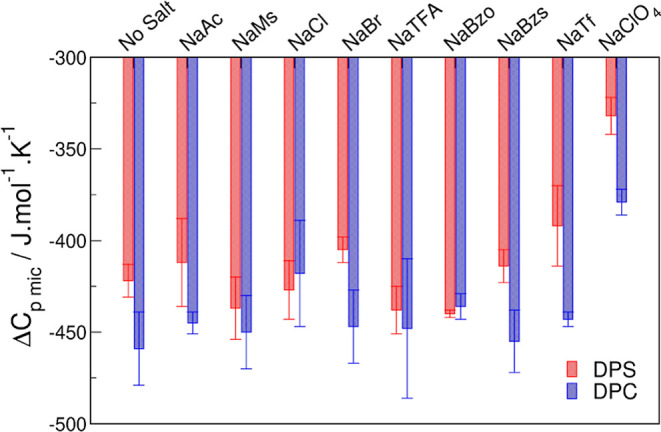
Heat capacity changes for the micellization of DPS (■, red) and DPC (■, blue) with 0.1 M NaX.

Linear fittings of the temperature dependence of Δ*H*_mic_^°^ for DPS and DPC in water, as examples, are given in Figure S1, Δ*C*_p,mic_ for all micelles systems are shown in Figure 5, and a table with all Δ*C*_p,mic_ values is given in Supporting Information (Table S3).

In general, heat capacity changes for DPC micellization were more negative than those for DPS, suggesting that more dehydration occurs during DPC micellization. Salt addition did not change Δ*C*_p,mic_ significantly, except for NaClO_4_. Our early work showed that the hydration of the DPS micelles is very similar when either of the salts is added (NaBr, NaMs, NaTFA, and NaTf)^34^ showing that dehydration of monomers is very similar for DPS containing these salts. However, the lower value of hyperfine splitting (*a*_N_) of the EPR spin label at the DPS micelle interface in NaClO_4_ (Supporting Information, Table S4 and Figure S2) shows that DPS micellar interfaces are less hydrated in the presence of NaClO_4_ than in water and other salts, contradicting the smaller magnitude Δ*C*_p,mic_ for NaClO_4_ determined here. For other charged surfactants, the use of Δ*C*_p,mic_ also fails to give insight into dehydration. For example, the counterion effect in [*C*_n_mim]^+^ could not be directly accessed by Δ*C*_p,mic_, since no direct correlation between the degree of counterion binding and Δ*C*_p,mic_ could be extracted, even when considering polar headgroup dehydration.^43^ And the Δ*C*_p,mic_ for *C*_n_TAC micelles with the increasing salt concentration also fails to give any insights into the micellization process.^62^

**Figure 6:**
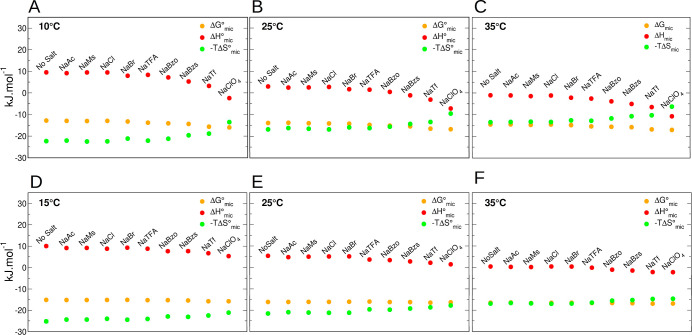
Gibbs energy, Δ*G*_mic_^°^ (●, yellow); enthalpy, Δ*H*_mic_° (●, red); and −*T*Δ*S*_mic_° (●, green) for surfactants in 0.1 M of NaX salts determined at different temperatures: DPS at (A) 10 °C, (B) 25 °C, and (C) 35 °C and DPC at (D) 15 °C, (E) 25 °C, and (F) 35 °C.

Eq 2 was used to calculate the standard Gibbs energy of micellization, and the entropy change was calculated from the standard micellization enthalpy and the standard Gibbs energy (eq 3). Δ*G*°, Δ*H*°, and *T*Δ*S*° of micellization for three temperatures are shown in Figure 6.

Calculation of the Gibbs free energy from CMC using the equation for ionic micelles was not used here. The approximation described by eq 2 is adequate because even at high salt concentrations, the maximum coverage of ClO_4_ anions in a similar propanesulfonate surfactant was only 0.2.^41^ However, we note that if the anions condensation were taken into account, the calculated Δ*G*° would be more negative for anions that adsorb more strongly to the micelle interface, like perchlorate and triflate, as observed in molecular dynamics simulations,^48^ and consequently, the new calculated variation of entropy (−*T*Δ*S*°) would be larger, i.e., more positive than those shown in Figure 6.

As noted for other surfactants,^10,62^ at lower temperatures the micellization Gibbs energy change is negative mainly due to the entropic contributions. At higher temperatures, however, it is primarily influenced by enthalpic contributions. Free energy of micellization changes for ZwS can be expressed as a sum of several free-energy contributions, i.e., the transfer of surfactant tails from water to the micellar core, packing, and deformation of the surfactant tails, headgroup steric interactions, formation of the aggregate–solvent interface, and headgroup dipole interactions.^65^ DPS and DPC micelles are both spherical and have similar aggregation numbers.^34,66^ Since the concentration of anions is higher in the region occupied by the ammonium propanesulfonate headgroup than in the layer composed of phosphocholine headgroup,^35^ the more significant effect observed on DPS micelles must be correlated to the anion penetration at the micellar core, causing dehydration of both the surfactant and the hydrotrope. The addition of ions should mainly affect the dipole term,^22^ so anion adsorption could explain the more negative Gibbs free energy, especially for DPS in NaClO_4_ and NaTf. From EPR results, it is possible to observe that the packing around the DPS headgroup area is also affected when NaClO_4_ is added due to lower mobility of the label in comparison to the systems with the other evaluated salts (Supporting Information, Figure S2 and Table S4). This would explain the smaller contribution of *T*Δ*S* to the Gibbs free energy of micellization for DPS in NaClO_4_.

## Conclusions

The addition of 0.1 M NaX permitted the thermodynamic analysis of the effect of anions on the micellization of zwitterionic DPS and DPC. Salt effects on the CMC and enthalpy of DPS and the enthalpy of DPC followed the order NaAc ∼ NaMs ∼ NaCl > NaBr > NaTFA > NaBzo > NaBzs > NaTf > NaClO_4_, showing that anion effect in zwitterionic micelle follows the same order as the anion effect in cationic micelles,^29,43^ although the effects are less pronounced. The salt effects depend on the surfactant dipole direction; the decrease in CMC and Δ*H*_mic_^°^ is more substantial for DPS, which has a positive charge closer to the micellar interface than DPC. The difference in anion adsorption at the zwitterionic micellar interface is known for a limited number of anions. However, here, we show that the effect is general and includes hydrotropic salts. The location of the positive charge in DPS, i.e., closer to the micellar core, leads to a stronger anion effect in the CMC than that observed in DPC, probably due to the possibility of these anions dehydrating themselves and the micellar interface upon binding closer to the hydrophobic portion of surfactant. Again, these effects are similar to those observed in cationic micelles, although in minor extension. It is also noteworthy that, similar to DPS, the effect of hydrotropic anions on DPC enthalpy of micellization also depends on the hydrophobic moiety of the anion,^48^ showing that this portion of the anions is pivotal to the thermodynamics of micellization even in zwitterionic systems. Considering the previous results from cationic systems^29^ this result seems to be general. We have also previously shown that dehydration is important for cationic peptide binding to mimetic membranes,^66^ showing that dehydration is a common and crucial effect for the adsorption of molecules at interfaces, regardless of the nature of the molecule and the interface. Finally, from the heat capacity changes, it can be argued that the micelles formed in salt solution are similar to those formed in water, except for NaClO_4_.

## Supporting Information Available

Tables containing thermodynamics data for all samples measured, linear fitting of Δ*H*_mic_ vs temperature for DPS and DPC in water, Δ*C*_p,mic_ and *T*_0_ for DPS and DPC in the presence of 0.1 M NaX, and EPR methods and results (PDF)

## Abbreviations

DPC: *N*-dodecylphosphocholine
DPS: *N*-dodecyl-*N*,*N*-dimethyl-3-ammonio-1-propanesulfonate
CMC: critical micelle concentration
ITC: isothermal titration calorimetry
EPR: electron paramagnetic resonance
Bzo: benzoate
Ac: acetate
Ms: methanesulfonate
TFA: trifluoroacetate
Tf: trifluoromethanesulfonate
Bzs: benzenesulfonate
5-MeSL: methyl 5-doxyl-stearate
a_N_: hyperfine splitting
