# Supplementary material for: Micellization of Zwitterionic Surfactant with Opposite Dipoles is Differently Affected by Anions
Source: Langmuir. 2025 Mar 31;41(14):9480–7. doi: 10.1021/acs.langmuir.5c00360 (PMC12004915; doi:10.1021/acs.langmuir.5c00360)
Supplement: Supplementary file 1 — la5c00360_si_001.pdf [file la5c00360_si_001.pdf]

Micellization of zwitterionic surfactant with opposite dipoles is differently affected by anions

Laura Mortara†#, Matheus P. Cortez†#, Caroline D. Lacerda†, Gustavo P. B. Carretero†, Shirley Schreier†, Hernan Chaimovich† Filipe S. Lima‡ and Iolanda M. Cuccovia† **

† Instituto de Química, Universidade de São Paulo, São Paulo 05513-970, Brazil

‡ Departamento de Química Fundamental, Centro de Ciências Exatas e da Natureza, Universidade Federal de Pernambuco, Recife 50670-901, Brazil

*(F.S.L.) E-mail: filipe.slima2@ufpe.br

*(I.M.C.) E-mail: imcuccov@iq.usp.br

#first authors

Table S1. DPS micellization parameters

Temperature / °C	cmc / mmol/L	ΔG° / $KJ.mol^{-1}$	ΔH° / $KJ.mol^{-1}$	TΔS° / $KJ.mol^{-1}$

No Salt				
10	4.30 ± 0.05	-12.83 ± 0.02	9.49 ± 0.11	22.32 ± 0.11
25	3.69 ± 0.14	-13.90 ± 0.10	2.97 ± 0.16	16.25 ± 0.18
35	3.41 ± 0.05	-14.55 ± 0.02	-1.05 ± 0.01	13.5 ± 0.02
NaAc				
10	4.06 ± 0.05	-12.97 ± 0.01	9.11 ± 0.29	22.08 ± 0.30
25	3.85 ± 0.13	-13.80 ± 0.08	2.45 ± 0.16	16.25 ± 0.18
35	3.48 ± 0.11	-14.51 ± 0.08	-1.11 ± 0.05	13.39 ± 0.10
NaMs				
10	3.91 ± 0.05	-13.05 ± 0.03	9.44 ± 0.37	22.50 ± 0.38
25	3.51 ± 0.10	-14.03 ± 0.07	2.74 ± 0.09	16.56 ± 0.11
35	3.19 ± 0.05	-14.72 ± 0.03	-1.44 ± 0.05	13.29 ± 0.05
NaCl				
10	4.05 ± 0.14	-12.97 ± 0.08	9.47 ± 0.35	22.44 ± 0.37
25	3.37 ± 0.10	-14.13 ± 0.06	2.74 ± 0.27	16.87 ± 0.10
35	3.41 ± 0.17	-14.55 ± 0.12	-1.15 ± 0.07	13.40 ± 0.14
NaBr				
10	3.58 ± 0.05	-13.26 ± 0.03	7.9 ± 0.30	31.17 ± 0.30
25	3.21 ± 0.06	-14.25 ± 0.05	1.69 ± 0.04	15.94 ± 0.10
35	3.04 ± 0.05	-14.85 ± 0.02	-2.19 ± 0.03	12.67 ± 0.10
NaTFA				
10	2.84 ± 0.05	-13.81 ± 0.04	8.32 ± 0.18	22.13 ± 0.18
25	2.61 ± 0.06	-14.77 ± 0.05	1.50 ± 0.07	16.27 ± 0.10
35	2.43 ± 0.05	-15.62 ± 0.06	-2.58 ± 0.03	12.84 ± 0.10
NaBzo				
10	2.51 ± 0.07	-14.09 ± 0.07	7.14 ± 0.25	21.24 ± 0.26

25	2.25 ± 0.11	-15.14 ± 0.13	0.48 ± 0.08	15.62 ± 0.15
35	2.25 ± 0.07	-15.62 ± 0.08	-3.86 ± 0.15	11.75 ± 0.17
NaBzs				
10	2.23 ± 0.05	-14.37 ± 0.06	5.26 ± 0.08	19.63 ± 0.10
25	1.95 ± 0.05	-15.49 ± 0.05	-1.14 ± 0.04	14.35 ± 0.10
35	2.10 ± 0.13	-15.79 ± 0.16	-5.06 ± 0.38	10.73 ± 0.32
NaTf				
10	1.30 ± 0.06	-15.65 ± 0.10	3.22 ± 0.56	18.87 ± 0;57
25	1.28 ± 0.10	-16.54 ± 0.18	-3.11 ± 0.24	13.44 ± 0.30
35	1.41 ± 0.05	-16.81 ± 0.07	- 6.51 ± 0.37	10.30 ± 0.37
$NaClO_4$				
10	1.12 ± 0.05	-15.99 ± 0.10	-2.43 ± 0.35	13.56 ± 0.36
25	1.16 ± 0.05	-16.77 ± 0.10	-7.21 ± 0.20	9.56 ± 0.22
35	1.29 ± 0.05	-17.05 ± 0.10	-10.76 ± 0.40	6.29 ± 0.41

Table S2. DPC micellization parameters

Temperature / °C	cmc / mmol/L	$\Delta G°$ / $KJ.mol^{-1}$	$\Delta H°$ / $KJ.mol^{-1}$	$T\Delta S°$ / $KJ.mol^{-1}$
No Salt				
15	1.85 ± 0.04	-15.08 ± 0.05	9.64 ± 0.01	24.72 ± 0.06
25	1.48 ± 0.01	-16.17 ± 0.02	5.40 ± 0.26	21.57 ± 0.29
35	1.60 ± 0.08	-16.50 ± 0.14	0.46 ± 0.08	16.96 ± 0.05

NaAc				
15	1.73 ± 0.04	-15.24 ± 0.06	9.14 ± 0.15	24.38 ± 0.19
25	1.49 ± 0.06	-16.16 ± 0.10	4.80 ± 0.27	20.96 ± 0.36
35	1.65 ± 0.08	-16.42 ± 0.13	0.24 ± 0.07	16.66 ± 0.06
NaMs				
15	1.74 ± 0.04	-15.23 ± 0.05	9.16 ± 0.26	24.39 ± 0.30
25	1.50 ± 0.07	-16.14 ± 0.12	5.01 ± 0.08	21.14 ± 0.19
35	1.50 ± 0.08	-16.67 ± 0.13	0.16 ± 0.04	16.83 ± 0.18
NaCl				
15	1.76 ± 0.04	-15.20 ± 0.04	8.81 ± 0.08	24.00 ± 0.04
25	1.51 ± 0.11	-16.12 ± 0.18	5.12 ± 0.31	21.24 ± 0.49
35	1.55 ± 0.03	-16.58 ± 0.06	0.41 ± 0.05	17.01 ± 0.10
NaBr				
15	1.78 ± 0.02	-15.16 ± 0.03	9.27 ± 0.03	24.43 ± 0.06
25	1.54 ± 0.03	-16.07 ± 0.05	5.15 ± 0.04	21.22 ± 0.07
35	1.55 ± 0.03	-16.54 ± 0.08	0.36 ± 0.05	16.89 ± 0.13
NaTFA				
15	1.69 ± 0.01	-15.29 ± 0.02	8.80 ± 0.06	24.09 ± 0.04
25	1.60 ± 0.04	-15.98 ± 0.07	3.66 ± 0.16	19.63 ± 0.23
35	1.58 ± 0.05	-16.64 ± 0.11	-0.15 ± 0.08	16.49 ± 0.03
NaBzo				
15	1.69 ± 0.00	-15.29 ± 0.00	7.64 ± 0.62	22.93 ± 0.62
25	1.47 ± 0.05	-16.20 ± 0.11	3.40 ± 0.30	19.77 ± 0.01
35	1.52 ± 0.06	-16.64 ± 0.02	-1.08 ± 0.19	15.57 ± 0.21
NaBzs				
15	1.59 ± 0.06	-15.45 ± 0.10	7.65 ± 0.70	23.09 ± 0.60

25	1.44 ± 0.02	-16.24 ± 0.05	2.79 ± 0.34	19.22 ± 0.20
35	1.44 ± 0.02	-16.77 ± 0.04	-1.47 ± 0.04	15.31 ± 0.00
NaTf				
15	1.38 ± 0.02	-15.79 ± 0.04	6.69 ± 0.11	22.48 ± 0.15
25	1.30 ± 0.02	-16.50 ± 0.04	2.18 ± 0.17	18.68 ± 0.21
35	1.30 ± 0.02	-17.04 ± 0.04	-2.19 ± 0.02	14.85 ± 0.06
$NaClO_4$				
15	1.38 ± 0.02	-15.79 ± 0.04	5.53 ± 0.26	21.13 ± 0.22
25	1.37 ± 0.04	-16.37 ± 0.06	1.44 ± 0.01	17.81 ± 0.05
35	1.37 ± 0.0	-16.89 ± 0.00	-2.23 ± 0.08	14.66 ± 0.08

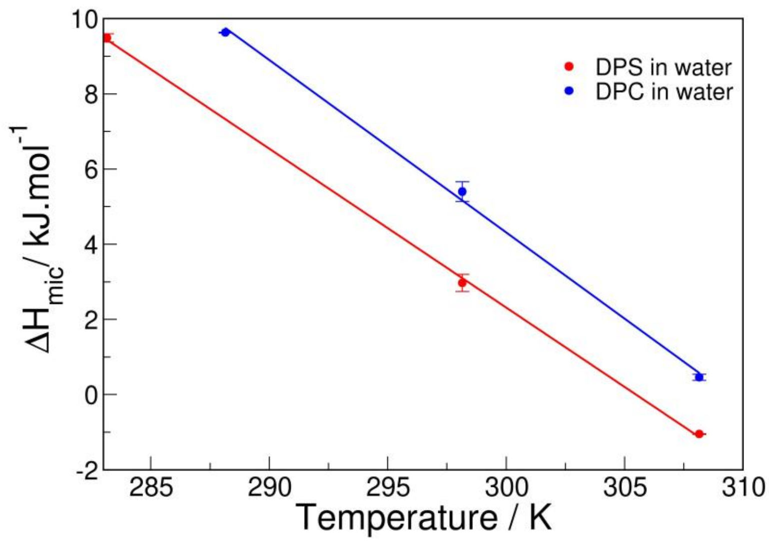

Figure S1. Linear fitting of ΔH_{mic} vs temperature for DPS and DPC in water

Table S3. $\Delta C_{p\ mic}$ and T_0 for DPS and DPC in presence of 0.1 M NaX

	DPS		DPC	
Salt	$-\Delta C_{p\ mic}$ / $J.mol^{-1}.K^{-1}$	T_0 / K	$-\Delta C_{p\ mic}$ / $J.mol^{-1}.K^{-1}$	T_0 / K
-	422 +/- 9	305	459 +/- 20	309
NaAc	412 +/- 24	305	445 +/- 6	309
NaMs	437 +/- 17	304	450 +/- 20	309
NaCl	427 +/- 16	305	418 +/- 29	310
NaBr	405 +/- 7	302	447 +/- 20	310
NaTFA	438 +/- 13	302	448 +/- 38	307
NaBzo	440 +/- 2	300	436 +/- 7	306
NaBzs	414 +/- 9	295	455 +/- 17	305

NaTf	392 +/- 22	291	443 +/- 4	303
$NaClO_4$	332 +/- 10	276	379 +/- 7	302

Electron Paramagnetic Resonance

Electron Paramagnetic Resonance (EPR). Measurements were obtained in a Bruker EMX-200 spectrometer, measured at a frequency of approximately 9.4 GHz, with a sweep width of 100 G, a modulation amplitude of 1.0 G, and a time constant of 0.163 s. Samples were added to flat quartz cells. Methyl 5-Doxyl-stearate and surfactants were added from chloroform stock solutions and the solvent was evaporated under N_2, and dried under vacuum for at least 2h. The solid was resuspended with the desired salt solution.

Spectra were analyzed with WINEPR software (Bruker), and the rotational correlation times (τ_B and τ_C) of the spin label were calculated from spectral line heights and line widths the equations below:[1]

$$\tau_b = -1.27x10^{-9}\frac{W_0}{2}\left[\left(\frac{h_0}{h_{-1}}\right)^{\frac{1}{2}} - \left(\frac{h_0}{h_{+1}}\right)^{\frac{1}{2}}\right] \qquad (1)$$

$$\tau_c = -1.19x10^{-9}\frac{W_0}{2}\left[\left(\frac{h_0}{h_{-1}}\right)^{\frac{1}{2}} - \left(\frac{h_0}{h_{+1}}\right)^{\frac{1}{2}} - 2\right] \qquad (2)$$

And the effective rotational correlation time is defined by[2]

$$\tau_{measured} = \sqrt{\tau_b x \tau_c} \qquad (3)$$

The effective rotational correlation time ($\tau_{measured}$) was determined from the spectra of 5-MeSL label inserted in DPS and DPC micelles in different salt solutions.[1,3] Examples of 5-MeSL spectra in DPS or DPC in 0.1 M $NaClO_4$ are shown in Figure 7, and $\tau_{measured}$ for all systems are in Table 1.

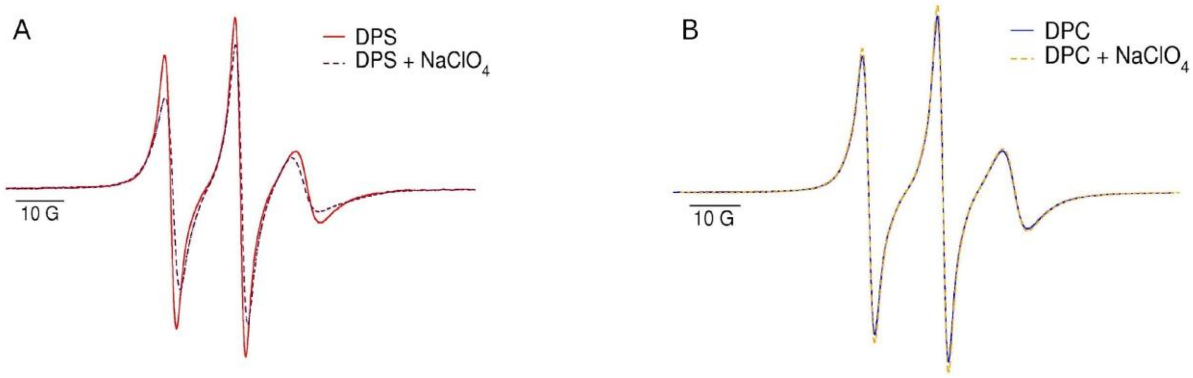

Figure S2. EPR spectra for 5-MeSL in DPS (A) or DPC (B) 0.02 M micelles in water (— for DPS and — for DPC) and $NaClO_4$ 0.1 M (··· for DPS and ··· for DPC) solutions.

Table S4. The values of $\tau_{measured}$ and hyperfine splitting (a_N) for 5-MeSL label incorporated into DPS and DPC micelles (0.02 M) in water or NaX 0.1 M solutions.

	DPS		DPC	
Salt	$\tau_{measured}$ /10^{-9} s	a_N / G	$\tau_{measured}$ /10^{-9} s	a_N / G
-	1.69	14.66	1.54	14.61
NaBr	1.79	14.56	1.52	14.66
NaBzs	1.68	14.52	1.40	14.61
NaTf	1.78	14.12	1.50	14.71
$NaClO_4$	2.04	14.27	1.56	14.61

The spectra in Figure S2 show narrow lines, indicating a fast tumbling of the probe on the EPR time scale. Especially in the high field line, one can observe a difference between both surfactants, with the broadening of lines for the label in DPS indicating a slower tumbling of the incorporated probe compared to the DPC micelles. From the $\tau_{measured}$, we can observe higher values for DPS micelles, specifically higher with $NaClO_4$, suggesting a higher local viscosity at the interface of the DPS micelles. For a surfactant with the same headgroup as DPS, the addition of high concentrations of $NaClO_4$ (1 M) led to a more compact interface.[4] Hyperfine splitting values, a_N, also point to a more dehydrated interface in DPS + $NaClO_4$.

References

(1) Ernandes, J.; Schreier, S.; Chaimovich, H. Spin Label Studies of Micellar and Pre-Micellar Aggregates. *Chem. Phys. Lipids* **1976**, *16* (1), 19–30. https://doi.org/10.1016/0009-3084(76)90012-8.

(2) Bales, B. L.; Zana, R. Characterization of Micelles of Quaternary Ammonium Surfactants as Reaction Media I: Dodeclytrimethylammonium Bromide and Chloride. *J. Phys. Chem. B* **2002**, *106* (8), 1926–1939. https://doi.org/10.1021/jp013813y.

(3) Tedeschi, A. M.; Franco, L.; Ruzzi, M.; Paduano, L.; Corvaja, C.; D'Errico, G. Micellar Aggregation of Alkyltrimethylammonium Bromide Surfactants Studied by Electron

Paramagnetic Resonance of an Anionic Nitroxide. *Phys. Chem. Chem. Phys.* **2003**, *5* (19), 4204. https://doi.org/10.1039/b305324p.

(4) Ferreira, G. S. S.; Périgo, D. M.; Politi, M. J.; Schreier, S. Effect of Anions from the Hofmeister Series and Urea on the Binding of the Charged and Uncharged Forms of the Local Anesthetic Tetracaine to Zwitterionic Micelles*. *Photochem. Photobiol.* **1996**, *63* (6), 755–761. https://doi.org/10.1111/j.1751-1097.1996.tb09627.x.
